# Are childhood oral health behaviours and experiences associated with dental anxiety in adolescence?

**DOI:** 10.1111/ipd.13058

**Published:** 2023-02-23

**Authors:** Jennifer Clow, Kate Northstone, Constance Hardwick, Mark Dermont, Tom Dudding

**Affiliations:** ^1^ Dental Public Health, Defence Primary Healthcare (Dental), HQ Defence Medical Services Group Lichfield UK; ^2^ Population Health Sciences, Bristol School of Medicine University of Bristol Bristol UK; ^3^ National Institute for Health and Care Research ACF, Dental Core Trainee, Bristol Dental School University of Bristol Bristol UK; ^4^ Head of Defence Public Health Unit, Consultant in Public Health, Defence Public Health Unit, Headquarters Defence Medical Services Group Lichfield UK; ^5^ National Institute for Health and Care Research ACF Restorative Dentistry, Honorary Lecturer Bristol Dental School, University of Bristol Bristol UK

**Keywords:** adolescence, ALSPAC, behaviours, childhood, dental anxiety, experiences

## Abstract

**Background:**

Dental anxiety is associated with untreated dental caries. Understanding which childhood behaviours or experiences have the strongest association with later dental anxiety may help focus preventive strategies, subsequently limiting the burden of dental caries and anxiety.

**Aim:**

The aim of this study was to explore whether behaviours and experiences during childhood were associated with adolescent dental anxiety.

**Design:**

Data were obtained from the Avon Longitudinal Study of Parents and Children (ALSPAC). Multivariable logistic regression was used to explore associations between adolescent dental anxiety and childhood behaviours and experiences. 1791 participants answered questions about oral health behaviours and experiences at 8 years of age and dental anxiety questions aged 17 years.

**Results:**

Children with experience of invasive dental treatment were more likely to have dental anxiety at 17 years of age than those who had not experienced dental treatment (OR 1.63; 95% CI: 1.12, 2.37; *p* = .011). Irregular dental attenders in childhood had over three times the odds of dental anxiety by adolescence, compared with regular attenders (OR 3.67 95% CI: 1.52, 8.88; *p* = .004).

**Conclusions:**

Adolescent dental anxiety is associated with invasive treatment and irregular dental attendance in childhood. A history of irregular attendance or invasive treatment may serve as a useful predictor when considering dental anxiety in young adult patients. Early preventive care supports good attendance and oral health. These actions may have secondary effects of reducing future dental anxiety.

**Why this paper is important to paediatric dentists**

- Regularly attending dental appointments to experience positive noninvasive treatments for caries prevention may also be protective against dental anxiety in adolescence.
- Considering behaviours and experiences in childhood, irregular dental attendance and invasive dental procedures could increase the risk of developing dental anxiety.
- Females and patients with a low socioeconomic status have a greater likelihood of reporting dental anxiety.

## 1 INTRODUCTION

In the United Kingdom, dentally anxious individuals experience more untreated dental caries, have poorer oral health‐related quality of life^1^ and face barriers accessing care.^1^ Dental caries is a preventable oral health problem affecting around half of the children in the United Kingdom,^2^ and dental anxiety exacerbates the burden of caries as these individuals are less likely to attend treatment and to receive preventive care.^3^ Pharmaceutical and behavioural treatments for dental anxiety are resource heavy.^3^ Strategies that can improve attendance and that focus on prevention could therefore have multifaceted benefits for oral health and well‐being and could reduce the burden on the health system.

Dental anxiety can range from feelings of unease to great distress and terror associated with dental settings, experiences or thoughts. Globally, 15.3% of adults and 10.0%–29.3% of children are estimated to be dentally anxious.^4^, ^5^ A high prevalence of dental anxiety has been reported in younger adults and females.^4^

Psychological theories propose that mechanisms for developing dental anxiety may be through dentally related direct conditioning,^6^ for example an unpleasant or painful experience, or indirectly through vicarious learning, learning from others.^6^ An extension to these theories, however, also suggests frequent and positive experiences of dentistry prevent dental anxiety, despite occasional painful experiences (termed latent inhibition).^6^, ^7^

The vicious circle of the dental fear model^8^ suggests that dental anxiety causes irregular dental attendance. Bidirectional causality, however, is plausible. Therefore, targeting attendance patterns could be an opportunity to prevent, rather than treating, dental anxiety.

In adults, a clear association exists between increased levels of dental anxiety and poorer oral health.^1^ This relationship is less certain in children;^9^, ^10^, ^11^ therefore, childhood and adolescence is a key period to explore associations between oral health and dental anxiety.

Dental anxiety is complex, subjective and personal with a multifactorial aetiology, which has been postulated to include attendance pattern,^9^ general anxiety levels,^12^ socioeconomic status^1^ and familial influences.^13^ Much of this understanding comes from cross‐sectional studies, which are subject to recall bias.^3^ A prospective longitudinal design, however, provides an opportunity to strengthen existing evidence of associations between dental anxiety and specific behaviours and experiences.

The aim of this prospective longitudinal study was to explore whether behaviours and experiences during childhood were associated with dental anxiety in adolescence. Objectives were to assess whether dental anxiety in adolescence was associated with specific suboptimal oral health behaviours or experiences in childhood and to assess the impact of sex and maternal education as confounders.

## 2 MATERIALS AND METHODS

Pregnant women in a geographic area in and around Bristol, with expected delivery dates from 1 April 1991 to 31 December 1992, were invited to join the Avon Longitudinal Study of Parents and Children (ALSPAC).^14^, ^15^ 14 541 pregnancies were enrolled initially resulting in 13 988 children who were alive at 1 year of age. After approximately 7 years, further eligible families, not initially enrolled, were recruited and the baseline group of children alive at 1 year of age was 14 901. Data were collected in multiple ways, including self‐completion questionnaires and through face‐to‐face visits. The study website contains details of data available, through a searchable data dictionary (http://www.bristol.ac.uk/alspac/researchers/our‐data/).

Ethics approval for the study was obtained from the ALSPAC Ethics and Law Committee and the local research ethics committees. Informed consent for the use of data collected via questionnaires and clinics was obtained from participants following the recommendations of the ALSPAC Ethics and Law Committee at the time.

**FIGURE 1:**
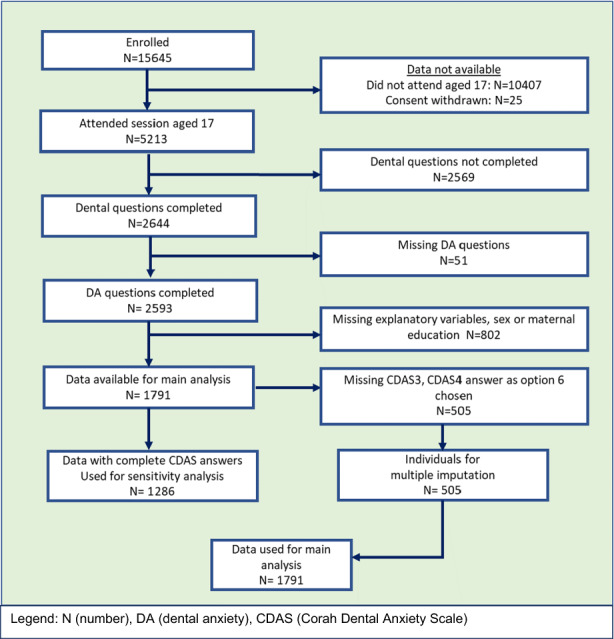
Flow diagram of participants available for analysis.

Dental questionnaires completed by ALSPAC children at approximate ages of eight and 17 years provided prospective data for this analysis.^16^, ^17^ From a total of 15 645 cases enrolled, 7129 completed the questionnaire aged 8 years and 2644 completed a self‐report dental questionnaire at approximately 17 years. Of these, 1791 had complete data available for analysis (Figure 1).

### 2.1 Outcome variable

The Corah Dental Anxiety Scale (CDAS) was used to identify dental anxiety at 17 years.^18^ The CDAS is comprised of four questions relating to dental scenarios. Five options are available for each question, ranging from feelings of no anxiety (scoring 1) to extreme anxiety (scoring 5; Table 1). Total CDAS scores range from 4 to 20, the latter indicating the greatest possible dental anxiety.^18^ Total scores ≥13 indicate dental anxiety.^19^

**TABLE 1:** Multiple‐choice questions used to generate the dental anxiety outcome variable, based on the Corah Dental Anxiety Scale.

1. If you had to go to the dentist tomorrow, how would you feel about it?	1. I would look forward to it as a reasonable enjoyable experience 2. I wouldn't care one way or the other 3. I would be a little uneasy about it 4. I would be afraid that it would be unpleasant and painful 5. I would be very frightened of what the dentist might do
2. When you are waiting in the dentist office feel turn in the chair how do you feel?	1. Relaxed 2. A little uneasy 3. Tense 4. Anxious 5. So anxious that sometimes I breakout in a sweat or almost feel physically sick
3. When you are in the dentist's chair waiting while he gets the drill ready to begin working on your teeth, how do you feel?	1. Relaxed 2. A little uneasy 3. Tense 4. Anxious 5. So anxious that sometimes I breakout in a sweat or almost feel physically sick
	6. Never had treatment from the dentist treatment with a drill
4. You are in the dentist's chair to have your teeth cleaned. While you're waiting and the dentist is getting out the instruments which he will use to scrape your teeth around the gums, how do you feel?	1. Relaxed 2. A little uneasy 3. Tense 4. Anxious 5. So anxious that sometimes I breakout in a sweat or almost feel physically sick
	6. Never had teeth cleaned by dentist

*Note*: Multiple‐choice questions are based on the Corah Dental Anxiety Scale,^21^ with the exception of the sixth option for Questions 3 and 4 (highlighted in blue). Multiple imputation was used to include the 505 participants that chose Option 6 in the main analysis.

Dental questionnaires that included the four CDAS questions were completed, and total CDAS scores were calculated for each participant. These scores were dichotomised to create dentally anxious (≥13) and not dentally anxious (<13) groups, which generated the binary outcome variable.

In ALSPAC, there was an additional sixth option for Questions 3 and 4. This allowed participants to state they had never experienced the scenario, rather than a feeling related to that scenario (Table 1 provides details). Where participants answered using the sixth option (not included in the original CDAS questionnaire), multiple imputation was used to impute the score for that question, rather than removing these individuals from the analysis. The proportion of participants with dental anxiety was calculated excluding those individuals with imputed values.

### 2.2 Explanatory variables

At approximately 8 years of age, dental experiences and behaviours were collected in a postal questionnaire completed with parental help. Specific behaviours and experiences explored in this analysis included regularity of dental attendance, toothbrushing frequency, age of first attendance, reason for first attendance and types of dental treatment experienced.

As regards visiting the dentist, participants who selected ‘regularly (for check‐ups)’ were classed as regular attenders (optimal behaviour). Those who chose ‘only for toothache’, ‘not ever, really’ or ‘never’ options were classed as irregular attenders (suboptimal). This generated the binary attendance variable. Twice‐daily toothbrushing is a well‐established behaviour considered necessary to prevent oral disease.^20^ Participants were asked to write the number of times a day they brushed their teeth. Children were categorised as ‘less than twice daily’ brushers (suboptimal) if their answer was <2. Those who reported brushing twice or more were placed in the ‘twice or more daily’ group (optimal) for the toothbrushing variable.

Two binary variables of having or not having the experience of: ‘treatment awake’ and ‘treatment asleep’ were generated. Treatment awake refers to participants who received invasive dental treatment. This includes those who responded yes to ever having a filling or local anaesthetic (which is required for an extraction or filling) without the use of sedation or general anaesthetics (GA). This did not include noninvasive orthodontic or preventive treatments. Treatment asleep refers to children who reported ever having GA or sedation for dental treatment.

Children were asked how old they were when they first visited the dentist. This answer was dichotomised into an optimal group (first attendance under 4 years of age) and a suboptimal group (first attendance aged 4 years and above), to generate the ‘first attendance age’ variable. The variable ‘reason for first attendance’ at the dentist was created using three categories: for a ‘check‐up’, ‘with parents’ or ‘for toothache’. The latter was considered as the suboptimal category.

The co‐variants of sex and maternal education were also collected. Highest level of maternal education was collected during pregnancy and categorised as high (degree or A‐level), medium (O‐level) or low (Certificate of Secondary Education or vocational). In the United Kingdom, O‐levels were the statutory examinations taken by 16‐year‐olds and A‐levels were optional, more advanced examinations, taken at around 18 years of age.

### 2.3 Statistical analysis

Analyses were undertaken using Stata/MP 17. Initially, cohort demographics were compared between the total enrolled (with complete demographic data) and the total used in the main analysis and sensitivity analysis in order to identify possible areas of bias.

Multiple imputation was used to include 505 participants who chose the sixth option for CDAS Questions 3 or 4. Answers to CDAS Questions 1 and 2 were used as predictors. Multivariable normal regression using a repetitive Markov chain Monte Carlo method approximated the distribution of the missing values for individuals that chose the sixth option. The imputations occurred 10 times, and estimation commands were run which adjusted the coefficients and standard errors for the variability between the imputations during analysis. This maximised the sample size available for the main analysis (*N* = 1791).

Logistic regression was used to calculate unadjusted and adjusted (controlling for sex and maternal education) odds ratios (ORs), 95% confidence intervals (CI) and *p*‐values for dental anxiety. Participants were similar in age at both of the data collection points, so age was not adjusted for. Mean ages of completion were 7.68 (standard deviation [SD] 0.21) and 17.21 (SD 0.36) years for the exposure and outcome variables, respectively.

Participants with complete CDAS data (*N* = 1286) were included in sensitivity analyses to assess the impact of using multiple imputation.

## 3 RESULTS

The demographic characteristics of the 1791 participants in the complete analysis differed from those originally enrolled. Table 2 shows almost two‐thirds of those in the analysis were female and over half had a high maternal education. This contrasts with the roughly even split between sexes and across the three maternal education categories at enrolment.

**TABLE 2:** Demographic characteristics at enrolment and analysis.

	Enrolment		Main Analysis		Complete CDAS data for sensitivity analysis						*p*‐value	Incomplete CDAS data	
	*N* = 12 461		*N* = 1791		*N* = 1286		Dentally anxious		Not dentally anxious				
												*N* = 505	
Sex	*N*	%	*N*	%	*N*	%	*N*	%	*N*	%	<.001	*N*	%
Female	6027	48.4	1096	61.2	771	59.9	82	10.6	689	89.4		325	64.4
Male	6434	51.6	695	38.8	515	40.1	13	2.5	502	97.5		180	35.6
Maternal education											<.0019		
High	4397	35.3	938	52.4	676	52.6	39	5.8	637	94.2		262	51.9
Medium	4317	34.6	587	32.7	419	32.6	31	7.4	388	92.6		168	33.3
Low	3747	30.1	266	14.9	191	14.8	25	13.1	166	86.9		75	14.8
Total	**12 461**		**1791**		**1286**		**95**	**7.4**	**1191**	**92.6**		**505**	

Abbreviations: CDAS, Corah Dental Anxiety Scale; Incomplete CDAS data, Multiple imputation used to include in main analysis; N, Number.

There was a greater proportion of reported dental anxiety in females and those with low maternal education. Logistic regression (Table 3) showed strong evidence that males were much less likely to report dental anxiety compared with females (OR 0.21; 95% CI: 0.13, 0.36; *p* < .001). Teenagers with low maternal education were almost three times more likely to have dental anxiety than those with a high maternal education (OR 2.61; 95% CI: 1.66, 4.10; *p* = .002). As the outcome used in this study is relatively rare, the ORs presented in our results approximate the risk (prevalence) ratio.

**TABLE 3:** Association between dental anxiety and explanatory variables.

Explanatory variable	Unadjusted				Adjusted		*p*‐value
	*N*	OR	95% CI	*p*‐value	OR	95% CI	
Sex				<.001			
Female	1096	1					
Male	695	0.21	0.13, 0.36				
Maternal education				.002			
High	938	1					
Medium	587	1.37	0.91, 2.08				
Low	266	2.61	1.66, 4.10				
Attendance				.004			.004
Regular	1758	1			1		
Irregular	33	3.45	1.47, 8.12		3.67	1.52, 8.88	
Daily toothbrushing				.845			.873
≥twice	1478	1			1		
<twice	313	0.95	0.59, 1.53		0.96	0.59,1.56	
First attendance age				.068			.095
Under 4	1556	1			1		
4 and older	235	1.55	0.97, 2.49		1.51	0.93,2.46	
First attendance reason				.352			.273
Check‐up	943	1			1		
With parent	839	0.93	0.65, 1.34		1.02	0.71, 1.48	
Toothache	9	3.46	0.70, 16.95		3.95	0.73, 21.32	
Ever had dental treatment while awake				.004			.011
No Tx awake	1254	1			1		
Tx awake	537	1.70	1.18, 2.45		1.63	1.12, 2.37	
Ever had dental treatment while asleep				<.001			<.001
No Tx asleep	1636	1			1		
Tx asleep	155	3.32	2.11, 5.22		3.14	1.96,5.04	

Abbreviations: 95% CI, Confidence interval; Adjusted, For sex and maternal education; N, Number; OR, Odds ratio; Tx, Treatment.

### 3.1 Treatment experience

Of those in the treatment awake group, most (89.2%, *N* = 479) experienced fillings and the remainder (*N* = 58) experienced a filling, extraction or both. Participants with experience of treatment awake by the age of eight had almost twice the odds of dental anxiety in adolescence compared with children without experience of treatment awake (OR: 1.70; 95% CI: 1.18, 2.45; *p* = .004). These odds attenuated minimally (OR: 1.63; 95% CI: 1.12, 2.37; *p* = .01) after adjusting for sex and maternal education. All further ORs reported are adjusted for sex and maternal education. A greater association with dental anxiety was seen for participants who had experienced treatment asleep in childhood, compared with those that had not (OR: 3.14; 95% CI: 1.96, 5.04; *p* < .001; Table 3).

### 3.2 Attendance behaviour

Irregular attenders were over three times more likely to have dental anxiety in adolescence compared with those who attended regularly as children (OR: 3.67; 95% CI: 1.52, 8.88; *p* = .004).

### 3.3 Other variables

Less than twice‐daily toothbrushing and first attending with parents showed no evidence of an association with dental anxiety (OR: 0.96; 95% CI: 0.59, 1.56; *p* = .873 and OR: 1.02; 95% CI: 0.71, 1.48; *p* = .273, respectively; Table 3).

### 3.4 Sensitivity analysis

7.4% of adolescents, with complete CDAS data (*N* = 1286), were classified as dentally anxious (Table 2). Females and those with a low maternal education reported the greatest proportions of dental anxiety, 10.6% and 13.1%, respectively. Sensitivity analysis (*N* = 1286) suggested that results were similar for all variables, except dental attendance (Table 4). Compared with the main analysis, the attendance variable effect size was smaller, the 95% CI crossed the null and *p*‐value indicated weak evidence (OR: 2.72; 95% CI: 0.85, 8.76; *p* = .093).

**TABLE 4:** Impact of sensitivity testing on explanatory variables of interest and their association with dental anxiety.

	Adjusted sensitivity analysis (*N* =1286)			
Explanatory variable	*N*	OR	95% CI	*p*‐value
Attendance				
Regular	1264	1		
Irregular	22	2.72	0.85, 8.76	.093
Ever had dental treatment while awake				
No Tx awake	835	1		
Tx awake	451	1.61	1.05, 2.48	.029
Ever had dental treatment while asleep				
No Tx asleep	1167	1		
Tx asleep	119	2.95	1.71, 5.10	<.001

Abbreviations: 95% CI, 95% confidence interval; N, Number; OR, Odds ratio; Tx, Treatment.

## 4 DISCUSSION

We found that experience of invasive dental treatment under sedation or GA (treatment asleep) was associated with around three times greater likelihood of dental anxiety in adolescence in a UK cohort of children.

The requirement for dental GA (or sedation) in childhood is predominantly for the extraction of carious teeth.^21^ Therefore, experience of treatment asleep could indicate experience of tooth extraction. An observational study^13^ found an association between tooth extraction and dental anxiety, with a similar OR (3.5) to the association found here between treatment asleep and dental anxiety. Directional causality for dental anxiety, however, cannot be assumed since dental anxiety could be a key reason for providing treatment under GA. Many factors including clinical necessity, parental preferences and levels of cooperation (not necessarily dental anxiety) may have contributed to the treatment asleep intervention.

An association was also seen between dental anxiety and treatment asleep. Restorations (fillings) are a common type of invasive dental treatment for children,^22^ and 89.2% (*N* = 479) of the treatment awake group experienced fillings. This study, however, showed that the association between dental anxiety and treatment asleep was much stronger than the association with treatment awake, suggesting that childhood experience of extractions plays a bigger role than experience of fillings. This idea is supported by other studies.^13^, ^23^

This research and other studies have shown dental anxiety to be associated with treatment asleep, treatment awake, tooth extraction^23^ or a bad dental experience.^21^ An unpleasant dental experience could be considered an overarching description for these three explanatory variables. Future work could focus on unpleasant dental experiences, rather than specific treatment types, to assess the strength of association.

Children reporting irregular dental attendance were more likely to be dentally anxious teenagers. This finding agrees with existing observational studies, reporting that dental anxiety and irregular dental attendance are associated^9^, ^13^ and fits with the mechanism of latent inhibition described by psychological theory.^6^ For example, regular attendance for check‐ups or simple noninvasive preventive treatments (such as fluoride varnish application) provide an opportunity to build positive nonpainful experiences.

This cohort showed a greater prevalence of dental anxiety in females and those with low maternal education. Despite the strong association between dental anxiety and these demographic variables, their confounding effect on attendance behaviour or treatment experience association was minimal. This highlights the importance of attendance and treatment experience as explanatory variables, irrespective of demographics.

Limitations of this study include cohort attrition, lack of power for an explanatory variable (reason for attendance) and potential misclassification bias, which are discussed below.

Attrition in the cohort may have influenced the results due to altered demographic proportions. Females were over‐represented with a 20% increase in enrolment. Participants with a low maternal education were under‐represented by half since enrolment (Table 2). This attrition is important because the true proportion of dental anxiety in those with low maternal education was likely to be under‐represented. It is possible that severely dentally anxious individuals failed to complete the dental questions, resulting in further bias, towards the null, thereby diluting the association of relevant behaviours and experiences with dental anxiety. The UK‐wide prevalence^24^ of dental anxiety in 15‐year‐olds has been reported as 10% which is higher than the 7.4% in this study, suggesting an element of under‐representation.

The inferences that could be drawn from some explanatory variables were limited due to a lack of power. For example, there was weak evidence of any association between dental anxiety and those who first attended for toothache, with only a small number of participants reporting that they first attended for toothache (*N* = 9). There was an attenuation in the association between attendance and dental anxiety in the sensitivity analysis. This is also likely to have been driven by a reduction in power.

The multivariable logistic regression was limited to confounders of sex and maternal education. This avoided using explanatory variables incorrectly as confounders, when these variables were acting as effect modifiers.

Despite successful validation,^19^ the CDAS has shortcomings that may have led to misclassification bias. Some clinicians argue that due to the order and phrasing of multiple‐choice questions, moderately dentally anxious individuals are not accurately identified and only highly anxious or very relaxed patients can be categorised accurately.^25^ Furthermore, CDAS does not include any questions related to local anaesthetic, which is a specific contributor to dental anxiety for some patients.^25^ Recall bias may have been present because the questionnaires asked the 8‐year‐olds and their parents to recall experiences that may have happened several years earlier. The dental experience questions though not formally validated in the ALSPAC questionnaire have been used in other studies, and when testing for sensitivity and specificity, a small risk of misclassification was found.^26^

Longitudinal studies can offer an opportunity to explore causality; as dental anxiety, however, was only measured at 17 years of age, it is not possible to confirm the temporality of relationships observed. It is possible that an individual developed dental anxiety after or during episodes of dental treatment during childhood or that it occurred after a period of irregular attendance but it is equally feasible that it pre‐dated these experiences.

This study contributes to evidence that irregular dental attendance and experience of invasive treatment in childhood are associated with a greater likelihood of dental anxiety in adolescence and these associations remained following adjustment for demographic variables. Other oral health behaviours and experiences, such as twice‐daily toothbrushing or first attending the dentist for a check‐up or with parents, appear to have no association with dental anxiety.

It is well established that childhood caries and subsequently dental treatment is a predictor for caries later in life.^27^ This study indicates that irregular attendance and dental treatment due to childhood caries may also be predictors of increased risk of dental anxiety by adolescence. Although the association with attendance should be treated with caution due to the sensitivity analysis, existing studies support this finding across several contexts.^3^, ^28^ The complex interplay of several potential co‐factors in inducing dental anxiety should be considered as part of future work aimed at generating hypotheses for testing. Work to develop a directed acyclic graph^29^ would be useful to help identify and explore the relationship between more factors. This would enable the correct inclusion of potential confounders and exclusion of effect modifiers and colliders in a more complex multivariable logistic regression based on larger sample sizes.

Childhood experience of dental care, including attendance patterns and the need for treatment, is associated with adolescent levels of dental anxiety. Besides considering a child's past experiences, sex and socioeconomic status, clinicians should also consider their influence on a young patient's feelings towards the dentist and how this may impact future morbidity and access to dental care. Where possible, positive treatment experiences should be provided; this, however, must be balanced with the clinical need for more invasive treatment to avoid the negative impacts of dental pain and infection and emphasises the need for early dental education.

This study has demonstrated associations between childhood attendance, previous dental treatment and adolescent dental anxiety in a large prospective cohort. These associations are based on self‐reported experiences and are not proof of causation; the findings, however, could serve as useful predictors for dental anxiety and help clinicians understand why some young adult patients are dentally anxious. Public health messaging around establishing early, regular childhood dental attendance to receive preventive focussed care remains important. It could also yield benefits in terms of reducing the risk of subsequent dental anxiety with all the individual impacts and treatment resource implications this entails.

## AUTHOR CONTRIBUTIONS

J. C. and T. D. conceived the ideas; M. D., T. D., and K. N developed ideas and the analysis plan; J. C. and C. H analysed the data; J. C. led the writing; all authors edited and reviewed the final document.

## CONFLICT OF INTEREST STATEMENT

The authors declare no conflict of interest.

## Data Availability

The data that support the findings of this study are available on request from the corresponding author. The data are not publicly available due to privacy or ethical restrictions.
